# Enhancing Cognitive Health in Elderly Individuals: The Impact of Hatha Yoga on Attention, Memory, and Reasoning: A Randomized Controlled Trial

**DOI:** 10.1155/jare/9990963

**Published:** 2025-09-08

**Authors:** Rania Oueslati, Mohamed Abdelkader Souissi, Sana Jarraya, Fatma Hilal Yagin, Georgian Badicu, Fahaid Al-Hashem, Luca Paolo Ardigò, Riadh Dahmen

**Affiliations:** ^1^Department of Human Sciences, High Institute of Sport and Physical Education Sfax, Sfax University, Sfax, Tunisia; ^2^Research Laboratory Education, Motricity, Sport and Health, LR19JS01, Sfax University, Sfax, Tunisia; ^3^Physical Activity, Sport and Health Research Unit, UR18JS01, National Observatory of Sport, Tunis, Tunisia; ^4^Department of Biostatistics, Faculty of Medicine, Malatya Turgut Ozal University 44210, Malatya, Türkiye; ^5^Department of Computer Science, Lakehead University, Thunder Bay P7B 5E1, Ontario, Canada; ^6^Department of Physical Education and Special Motricity, Faculty of Physical Education and Mountain Sports, Transilvania University of Braşov, Braşov 500068, Romania; ^7^Department of Physiology, College of Medicine, King Khalid University, Abha, Saudi Arabia; ^8^Department of Teacher Education, NLA University College, Oslo 0166, Norway

**Keywords:** attention, Hatha yoga, memorization, older adults, reasoning

## Abstract

**Background:** Aging leads to physiological and psychological changes that compromise both mental and physical autonomy, as well as cognitive functions, thereby increasing the risk of anxiety and depression. The sedentary lifestyle typical of older individuals results in a deterioration of the overall quality of life and well-being.

**Objective:** This study aims to evaluate the effectiveness of Hatha yoga in improving cognitive health among older adults. We will specifically examine the impact of this practice on attention, memory, and reasoning.

**Methods:** The present study assesses the impact of Hatha yoga on attention, memorization, and reasoning in healthy older adults aged between 65 and 80 years. The study population comprises 45 healthy individuals (26 men and 19 women; 72.3 ± 5.6 years) residing in a retirement home, divided into three groups: a yoga group (YOGA, *n* = 15) that participated in yoga sessions; a physical activity group (APS, *n* = 15) engaged in sports and physical activities sessions; and a control group (CONT, *n* = 15) that did not undertake any activities. The study spanned 24 sessions, with two sessions per week lasting 45 min each. Participants completed test sessions dedicated to evaluating attention, memory, and reasoning before (T0) and after (T1) 12 weeks. A two-way ANOVA was used to analyze the differences between groups and over time.

**Results:** After the intervention sessions, the data showed that the YOGA group registered significantly greater improvements at T1 compared to that of T0 in all cognitive parameters (e.g., attention (*p* < 0.001, Hedges' *g* = 1.35), memory (*p* < 0.001, Hedges' *g* = 1.04), and reasoning (*p* < 0.001, Hedges' *g* = 1.82)). Furthermore, our results revealed a significant difference between the YOGA group and both the APS (*p* < 0.001) and CONT (*p* < 0.01) groups for the attention and reasoning parameters at T1.

**Conclusions:** This study underscores the potential of Hatha yoga to enhance the mental well-being of the elderly, suggesting significant benefits for cognitive well-being in this population.

**Trial Registration:** Pan African Clinical Trials Registry: PACTR202405804830163.

## 1. Background

Aging encompasses a myriad of physiological and psychological processes that modify the structure and functions of an organism over time [1]. This complex progression is marked by changes affecting physical structures, physiological functions, and cognitive activities, resulting in a loss of mental and physical autonomy and cognitive declines [2, 3]. These cognitive declines have profound implications for daily life and contribute to an increased risk of anxiety and depression [4].

Biologically, aging introduces changes in the central nervous system and increases the risk of conditions such as osteoarthritis [5]. The sedentary lifestyle often observed in older individuals is characterized by low energy frequency, leading to a decline in the quality of life and general well-being [6]. Aging further affects attentional capacities, leading to a decrease in visual perception, which impacts cognitive functions like problem-solving ability and manual precision [7]. Executive functions and responses to obstacles also deteriorate with normal aging [8]. Cognitive aging manifests as a decline in various cognitive abilities, including memory, attention, perception, reasoning, problem-solving, and information processing speed [9].

To counter the effects of aging on cognitive function, regular physical activity is proposed as a beneficial intervention [10]. Numerous studies suggested that lifelong exercise regimes can improve cognitive function, including attention, processing speed, executive function, and memory [11]. For example, a home-based program of balance and strength retraining significantly improved selective attention among seniors [12]. Other studies demonstrated that resistance training improved memory performance and physical and cognitive capacity in older adults [8, 13, 14]. Additionally, aerobic exercise has been linked to improved spatial memory performance [15].

Barnes et al. [16] confirm the positive correlation between the physical condition of individuals and their cognitive vitality. For instance, a comparison by Clarkson-Smith & Hartley [17] revealed that physically fit individuals aged between 55 and 91 responded more quickly in simple reaction time tasks than sedentary counterparts. Another study of de Bruijn et al. [18] found that women with higher levels of physical activity were less likely to have cognitive decline. Physical activity has been associated with improved cognitive functioning in seniors [19], and intervention programs aim to enhance the physical condition of the elderly to boost cognitive vitality [20].

Various studies have consistently shown that regular physical activity plays a crucial role in maintaining cognitive health and reducing the risk of cognitive decline in older adults [21, 22]. Physical activity is associated with improved attention, working memory, and executive functions, contributing to overall cognitive vitality [15]. Additionally, yoga, with its emphasis on breathing, meditation, and physical postures, is increasingly recognized as beneficial for seniors' health and cognitive well-being [23–25]. Studies have demonstrated that yoga interventions, such as Hatha yoga, can enhance memory and executive functions among older adults [26–29]. For instance, McDougall et al. [26] reported significant improvements in memory performance and self-efficacy following a yoga intervention. Furthermore, Morone and Greco [25] highlighted the positive impact of yoga on reducing pain and enhancing overall health in older adults.

In a second aspect, yoga has demonstrated its efficacy in enhancing attention, as supported by various studies. Sivakumar et al. [30] conducted a 6-month yoga intervention, revealing significant cognitive improvements in attention and processing speed. Another study on older adults found that the yoga group outperformed the stretching and strengthening training group in attention and processing speed, suggesting that yoga postures involve an active attentional component [31]. A meta-analysis also supported the positive impact of mind–body (MB) training, such as yoga and mindfulness, on attention, working memory, and global cognition [32]. Further evidence from Gothe et al. [31] indicated that an 8-week Hatha yoga program improved attentional and information processing abilities in older adults.

Moreover, a study by Gothe et al. [28] demonstrated beneficial effects of Hatha yoga on cognitive functions, including attention and memory. Kundalini yoga, as explored in a previous study, was associated with improved neural activity, brain structural alterations related to executive function, enhanced memory performance, awareness, and attention in seniors with mild cognitive impairment (MCI). Various forms of yoga, particularly Hatha yoga, which emphasizes physical postures (asanas), have consistently demonstrated positive benefits in enhancing sustained attention, memory, and executive functions [33]. Hatha yoga is chosen for its structured approach to postures and breathing techniques, which are believed to promote cognitive health through increased oxygenation and relaxation responses.

Concerning reasoning, limited studies have explored the relationship between yoga and reasoning in seniors. However, in younger populations, research has shown positive effects. For instance, Mondal and Kundu [34] conducted a 12-week yoga program on students aged 11–13, resulting in a significant improvement in reasoning ability. Additionally, mindfulness practices, which involve attention in the present moment, have been associated with positive effects on reasoning, as conscious awareness enables intentional choice [35].

Given the multifaceted nature of cognitive decline associated with aging, including domains such as attention, memory, and reasoning provides a comprehensive assessment of the potential benefits of Hatha yoga on cognitive health in older adults. While research on yoga and reasoning in seniors is relatively scarce, studies involving younger populations have been insightful [34]. Given the noble nature of research involving yoga and seniors, the primary objective of the present study is to investigate the effects of Hatha yoga practice on cognitive functions including attention, memory, and reasoning among older adults. The hypothesis posits that, compared to both the physical activity and control groups, the yoga group will exhibit significant improvements in attention, memorization, and reasoning.

## 2. Methods

### 2.1. Participants

**Figure 1:**
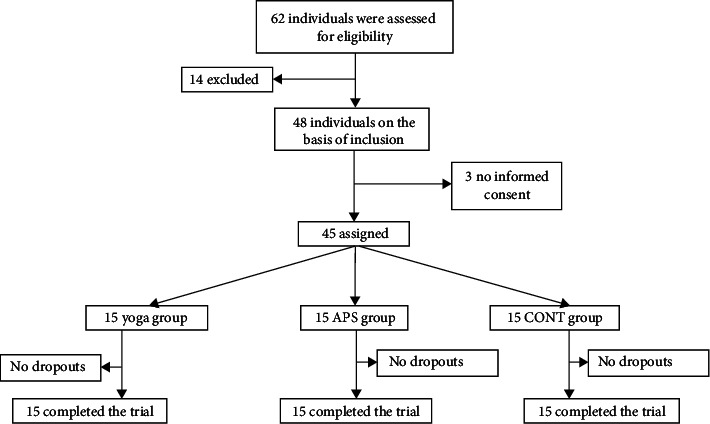
Flow diagram of the study.

G∗power software [36] was used to calculate the required sample size. A significance level (*α*) of 0.05 and a power of 0.80 were specified. After discussions among the authors, the effect size was estimated at 0.7 (medium effect). Consequently, a sample size of 54 participants was deemed necessary. However, due to practical constraints such as availability, only 45 older adults could be included. At the beginning of the experimentation, 62 older adults were selected as potential participants for this study. Fourteen of them were excluded because they did not meet the inclusion criteria, and three did not provide informed consent by signing the required form (Figure 1).

A total of 45 individuals, aged 65–80 years (26 men and 19 women), residing in a retirement home, voluntarily chose to participate in this study. It is a public retirement home located in an urban area in Sousse, Tunisia, with a large garden. This home offers various types of services such as medical care, recreational activities, wellness programs, and psychological support services. The establishment has diverse types of staff, including nurses, doctors, caregivers, and activity coordinators, who work together to ensure the well-being of the residents.

All participants were fully informed about the main objective and experimental procedures of the study. Moreover, they were clearly informed that their participation was voluntary, and they could withdraw at any time. Before joining the study, each participant provided a signed informed consent, indicating their acceptance to participate in the experiment.

To be included in the study, each participant had to meet the following criteria: (i) be aged between 65 and 80 years, (ii) have normal or corrected-to-normal vision (vision was assessed using standard optometric exams), and (iii) not have mental problems or a history of musculoskeletal disorders that could have affected their physical capacity.

After the pretest session, in accordance with the CONSORT guidelines, participants were randomly allocated using blinded allocation. The random allocation sequence was generated by an independent investigator using computer-generated random numbers. Subsequently, another researcher enrolled participants, ensuring that they were assigned to one of the three groups undergoing different interventions: (i) a yoga group (YOGA, *n* = 15; age = 72.12 ± 3.35 years) attended yoga sessions, (ii) a physical activity group (APS, *n* = 15; 73.25 ± 2.83 years) participated in sports exercises and physical activities, and (iii) a control group (CONT, *n* = 15; 74.4 ± 3.55 years) did not engage in any activity. Descriptive characteristics of these participants are presented in Table 1. The study was conducted following the guidelines of the Declaration of Helsinki (2013), and the experimental protocol was approved by the local Research Ethics Committee (CEFMS 148/2022).

### 2.2. Procedure

This experiment took place in a gymnasium located at the retirement home in Sousse, Tunisia, during the months of January, February, and March 2023. Before the start of this experimentation, a familiarization session with the experimental procedures was scheduled to minimize the impact of learning during the current study. Following this session, participants in each group underwent two testing sessions, dedicated to the assessment of attention, memory, and reasoning, before (T0) and after (T1), over 12 weeks, with two sessions per week, each intervention session lasting 45 min. All testing sessions were conducted in the morning between 9:30 a.m. and 10:15 a.m., under constant environmental conditions (21°C–23°C). In addition, no changes to trial outcomes or intervention protocols were made after the trial commenced.

During this experiment, a physical education teacher with 20 years of experience in the field, holding dual expertise in basketball and yoga, was involved in the conduct of this study.

At the beginning of each intervention session, participants from both experimental groups engaged in a general warm-up for the upper and lower limbs followed by stretching aimed at reducing the risk of bodily tension during the session. Subsequently, each participant followed a pre-established program lasting 30 min, tailored to the functioning mode of their group. Finally, we concluded each intervention session with 5 min of stretching. In fact, the groups were characterized as follows.

#### 2.2.1. Yoga Group (YOGA)

During the central part of the yoga sessions, participants dedicated themselves to practicing various yoga postures, also known as Asana. These postures included the seated mountain pose, seated chair twist, seated warrior position, goddess pose with a twist, pigeon poses, and seated forward bend pose. The breathing techniques, known as Pranayama, involved the voluntary regulation of breath, incorporating methods such as forceful exhalation with passive inhalation, rapid inhalation and exhalation, as well as slow and rhythmic alternate nostril breathing.

#### 2.2.2. Physical Activity Group (APS)

During the central part of the physical activity sessions, participants in the group engaged in low-intensity basketball exercises regularly. These exercises included the following elements: passes, dribbles, free throws, and fakes, organized in a circuit with size 5 basketballs (685–700 mm in circumference, 465–495 g). These balls were chosen for their lightness and flexibility, making them easier to grip and control while also reducing muscle fatigue and minimizing joint tension.

#### 2.2.3. Control Group (CONT)

During this experimental period, participants in this group did not engage in any type of physical activity and maintained their usual activities within the retirement home facility.

The participants were closely monitored throughout the study, and no adverse effects were reported in any of the intervention groups.

### 2.3. Data Collection

#### 2.3.1. Reasoning

**Figure 2:**
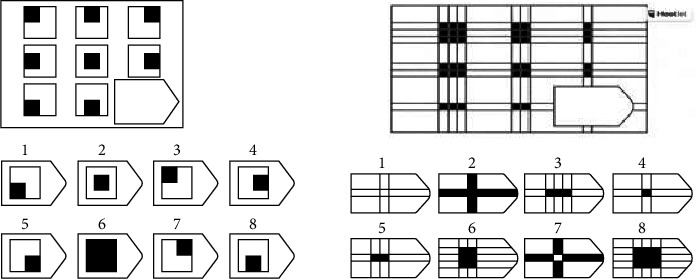
Excerpt from the Raven progressive matrices test.

To assess reasoning in older adults, we adopted the Raven progressive matrices test. In fact, Raven matrices, developed by Raven & Court [37], comprised a diverse set of intelligence tests. Originally designed for children aged 5–11 and adults aged 65 and older, this nonverbal intelligence test has been employed across various age groups for different research purposes (Figure 2).

The test involved presenting a series of incomplete geometric figures in the lower right-hand corner, challenging participants to decipher and organize the disorganized images. It assessed the ability to perceive abstract patterns, identify relationships between them, conceptualize the nature of the figures, and employ sound reasoning methods.

Executing the test demanded a high level of attention and reasoning, requiring individuals to adopt an exploratory and comparative approach and utilize multiple sources of information to complete the matrices. Participants were presented with six alternatives, from which they chose the one that best completes the pattern. Typically lasting around 15 min, the score reflects the number of correct answers.

#### 2.3.2. Memorization

**Figure 3:**
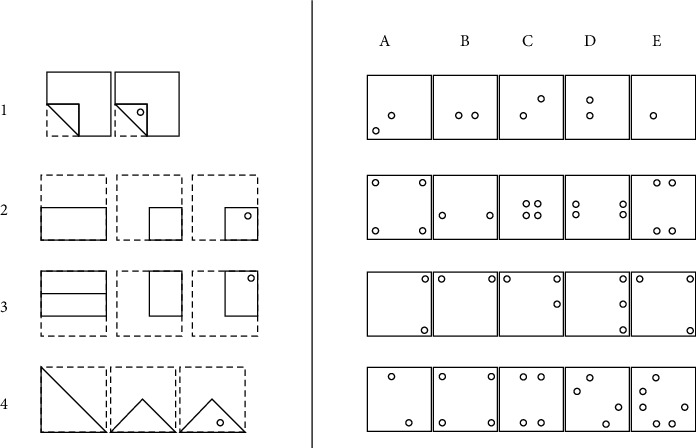
Excerpt from the paper folding test.

To assess memorization in older adults, we adopted the paper folding test. In fact, this test was applied as per the French et al.'s [38] kit of cognitive tests. It is constituted from 20 items divided into two sections of 10 items, each. The different items contained several folding steps. This test required participants to mentally perform complex spatial maneuvers. For each test item, successive drawings represented two or three folds on a square sheet of paper. The last drawing on the paper showed a hole that has been drilled. Elderly subjects had to choose one of the five drawings to show what the perforated paper would look like when completely reopened (score range from 0 to 20). The duration of the test was 6, 3 min for each page test. The score was calculated by the number of correct answers as described in Figure 3.

#### 2.3.3. Attention

To assess memorization in older adults, we adopted the Wais code test. In fact, this test was invented by the American psychologist Wechsler in 1955 [39]. It was one of the most used tests to evaluate the cognitive functioning [40] and to measure the cognitive abilities of adolescents, adults, or older adults aged from 16 to 89 years.

**Figure 4:**
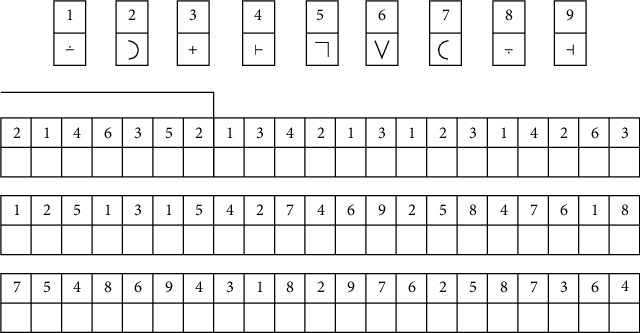
Excerpt from the WAIS code test.

In this test, each line was made up of two lines; the top line in which the numbers were written and the bottom line in which there were white boxes. Simply copy the signs corresponding to the numbers from 1 to 9 visible at the top of the page and copy them correctly under the correct numbers below (Figure 4). The goal is to go as far as possible and fill all the blank squares in a limited time as quickly as possible. The test took 90 s to finish, and the score reflected the number of correct answers.

### 2.4. Statistical Analysis

Statistical tests and analysis were performed by the *Statistica 10 software(StatSoft, Cracow, Poland),* The data from the current study were presented in the form of means and standard errors (mean ± SE) for the parameters of reasoning, memory, and attention.

The verification of the normality of the distribution of the studied parameters has been done using the Shapiro–Wilk test. In addition, the homogeneity of variance was tested (Levene test). The results were analyzed using a two-factor ANOVA (3 [Group: yoga; Aps and CONT] × 2 [time: T0 and T1]) with repeated measures on the last factor. For each analysis, when the ANOVA showed a significant effect, a Bonferroni post hoc test was applied to compare experimental data in pairs.

To assess practical relevance, effect sizes (*ηp*^2^) were calculated, with *ηp*^2^ ≥ 0.01 indicating small, ≥ 0.06 medium, and ≥ 0.14 large effects. The statistical significance level was set at *p* ≤ 0.05.

## 3. Results

**Figure 5:**
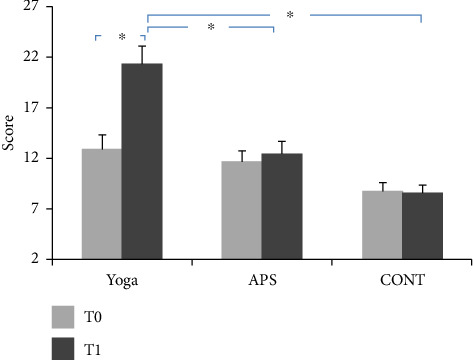
Representation of the evolution of attentional performance after 24 sessions in the three groups (mean ± standard deviation); APS: physical activity and sports group; CONT: control group; ^∗^significant difference compared to the APS and control groups (*p* < 0.001).

**Figure 6:**
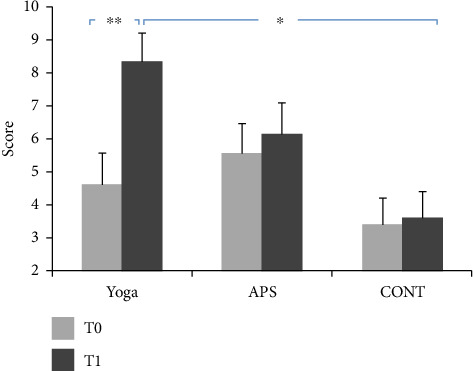
Representation of the evolution of memory performance after 24 sessions in the three groups (mean ± SD; APS: physical activity and sports group; CONT: control group; ^∗^, ^∗∗^significant difference (*p*=0.006 and *p* < 0.001, respectively).

**Figure 7:**
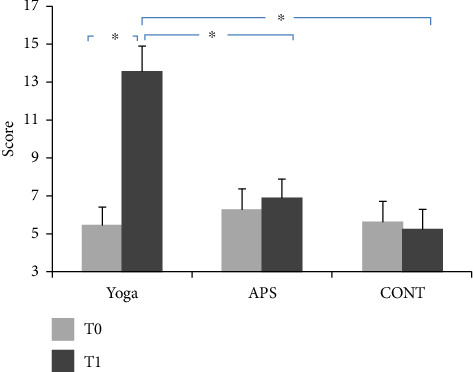
Representation of the evolution of reasoning performance after 24 sessions in the three groups (mean ± SD; *n* = 45); APS: physical activity and sports group; CONT: control group; ^∗^: significant difference compared to the APS and control groups (*p* < 0.001).

Descriptive statistics presented as means (±SE) are summarized in Figures 5, 6, 7. At baseline (pretest), single-factor ANOVA revealed no significant differences between groups for reasoning, memory, and attention variables (*p* > 0.05).

### 3.1. Attention

The average values of attention performance recorded during the pretest and post-test are presented in Figure 5.

The ANOVA showed a significant group effect (*F*(2, 42) = 11.96; *p* < 0.001; *ηp*^2^ = 0.36). The ANOVA statistical analysis also showed a significant time effect (*F*(1, 44) = 166.41; *p* < 0.001; *ηp*^2^ = 0.79). In addition, the ANOVA showed a significant interaction effect (group × time) (*F*(2, 42) = 133.45; *p* < 0.001; *ηp*^2^ = 0.86).

For the yoga group, compared with the pretest, the post hoc test showed higher values at the post-test for attention scores (*p* < 0.001, *g* = 1.35, 95% CI = −9.66 to −7.14). Additionally, the post hoc test revealed significantly higher values for the yoga group compared with those for the APS group and CONT group at the post-test for attention scores (*p* < 0.001, 95% CI = 3.37–14.23 and 95% CI = 7.23–18.1, respectively).

### 3.2. Memorization

The average values of memorization performance recorded during the pretest and post-test are presented in Figure 6.

The ANOVA showed a significant group effect (*F*(2, 42) = 3.13; *p* < 0.05; *ηp*^2^ = 0.12). The ANOVA statistical analysis also showed a significant time effect (*F*(1, 44) = 116.85; *p* < 0.001; *ηp*^2^ = 0.73). In addition, the ANOVA showed a significant interaction effect (group × time) (*F*(2,42) = 63.85; *p* < 0.001; *ηp*^2^ = 0.75).

For the yoga group, compared with the pretest, the post hoc test showed higher values at the post-test for memorization scores (*p* < 0.001, *g* = 1.04, %95 CI = −4.49 to −2.98). Additionally, the post hoc test revealed a significantly higher value for the yoga group compared with that for the CONT group at the post-test for the memorization scores (*p*=0.006, %95 CI = 0.81–8.65).

### 3.3. Reasoning

The average values of attention performance recorded during the pretest and post-test are presented in Figure 7.

The ANOVA showed a significant group effect (*F*(2,42) = 3.99; *p* < 0.03; *ηp*^2^ = 0.15). The ANOVA statistical analysis also showed a significant time effect (*F*(1, 44) = 133.87; *p* < 0.03; *ηp*^2^ = 0.76). In addition, the ANOVA showed a significant interaction effect (group × time) (*F*(2, 42) = 125.85; *p* < 0.001; *ηp*^2^ = 0.85).

In the yoga group, there were higher values in reasoning scores at the post-test compared to those at the pretest (*p* < 0.001, *g* = 1.82, %95 CI = −9.43 to −6.84), as indicated by the post hoc test. Moreover, significant differences were observed in reasoning scores at the post-test, with the yoga group showing higher values compared to both the APS and CONT groups (*p* < 0.001, 95% CI = 1.96–11.37 and 95% CI = 3.63–13.03, respectively) according to the post hoc test.

## 4. Discussion

The main objective of this study is to assess the impact of Hatha yoga intervention on various cognitive functions such as attention, memory, and reasoning among Tunisian subjects aged 65–80 years, compared to a group engaging in regular basketball physical activity and a sedentary control group.

The obtained results revealed a significant positive effect of yoga on the majority of parameters related to attention, memorization, and reasoning over the 12-week intervention period, compared to both the physical activity and control groups. Seniors participating in the yoga program improved their skills related to attention, as measured by the Wais code test [39], memorization, assessed by the paper folding test [38], and reasoning capacities, evaluated by the Raven progressive matrices [37], in comparison to the physical activity and control groups.

The improvement in attention through yoga in this study aligned with results demonstrating that Hatha yoga training can positively influence attention in the elderly. A study by Sivakumar et al. [30] aimed to explore the benefits of a 3-month yoga intervention on attention in older adults, revealing a significant improvement in the yoga group compared to the control group. This underscored the potential of yoga-based interventions in enhancing attentional skills in the elderly. Similarly, Alexander et al. [41] indicated that Hatha yoga enhances attention skills. In a related study examining the effects of an Iyengar yoga program on cardiovascular (CV) risk in older adults, practicing gentle yoga for 8 weeks demonstrated improvements in the overall physical function, stress reduction, anxiety alleviation, and attention improvement. These findings are consistent with previous research suggesting that yoga and meditation can mitigate attention deficits in Alzheimer's patients [42].

Moreover, our results align with previous studies, such as Gothe et al. [28, 31], indicating that yoga practice enhanced information processing speed and attentional abilities. The recent systematic reviews [43, 44] and meta-analysis by Gothe & McAuley [33] collectively concluded that yoga interventions exhibit positive effects on cognitive functions such as attention, memory, and executive functions across various populations, including older adults. In addition, it was revealed that yoga also improves psychological well-being [45], quality of life [24], and physical functioning [46]. Several studies suggested that yoga techniques may enhance cognitive function and overall health [47].

Within the context of the elderly, the study by Wiesmann et al. [48] examined the sense of coherence in older individuals, offering options such as endurance training, muscle strengthening, yoga classes, or meditation. The results indicated that systematic participation in an age-appropriate program, promoting an active and productive lifestyle, could contribute to an experience of life that fosters coherence and health in advanced age. Regarding cognitive abilities, Chan et al. [49] compared seniors practicing MB exercises (such as yoga and tai-chi) and CV exercises with those who did not exercise in a memory test. The MB and CV groups showed comparable results, surpassing the control group. Significantly, individuals practicing both MB and CV exercises achieved the best performance, showing no degradation compared to other groups.

Concerning reasoning function, the present study suggested a positive impact of a yoga program on this function in the older adults. However, to our knowledge, no studies have been conducted to date on the correlation between yoga and reasoning in this demographic. This contrasts with findings when studying the impact of yoga on youth. Indeed, our results align with studies involving yoga in youth, such as Mondal and Kundu [34]. This study, including two groups of students aged 11–13, demonstrated a significant improvement in reasoning ability after 12 weeks of yoga practice.

Our results also align with the findings of Travis et al. [50] and Shapiro [51], who discussed the effect of mindfulness on reasoning in seniors (MB practice). It is only through mindfulness that we have the possibility of conscious choice. In the same context, our results are coherent with those of Shapiro et al. [52], who conducted a study examining the effect of mindfulness on moral reasoning in adults, demonstrating that mindfulness can facilitate moral reasoning and decision-making.

MB interventions based on yoga for older adults seem to be a safe, feasible, and effective alternative for maintaining cognitive functions in both age-related and disease-related cognitive decline. Practicing yoga can be a useful part of the daily routine to maintain cognitive function in advanced age.

This study sheds significant light on the potential benefits of yoga on various aspects of cognitive functions in older individuals. The results suggested that yoga can improve attention, memorization, and reasoning, offering a holistic approach to maintaining and stimulating cognitive health in the elderly. These findings reinforce the idea that yoga, as a nonpharmacological intervention, can play a positive role in enhancing the mental quality of life for older individuals.

It is important to note that, while this study has highlighted encouraging results, further longitudinal research and randomized controlled trials are needed to consolidate these conclusions and better understand the specific mechanisms through which yoga exerts its beneficial effects. Additionally, exploring the long-term effects of yoga on the cognitive health of older individuals would be a valuable direction for future research.

Furthermore, it is crucial to consider alternative explanations or potential confounding factors that may influence the observed results. For instance, although the groups were initially balanced through randomization, other unmeasured variables such as participants' level of engagement in the different activities offered or lifestyle habits could have played a role. Additionally, while ophthalmological tests were used to assess normal or corrected-to-normal vision, individual variations in visual perception could also impact the measured cognitive performances.

### 4.1. Limitations

Our research has certain limitations that warrant consideration. One limitation of the current study is the limited number of participants, which could restrict the generalization of the obtained results. A larger and more diverse sample size would have enhanced the statistical power, thereby strengthening the robustness of data interpretation.

The duration of each yoga session in our study was set at 45 min, in contrast to the typical 1 h and 20-min duration reported by Lark [53]. While this adjustment was made to accommodate the attention span of older adults, it does constitute a limitation as it deviates from the standard yoga session duration. Future research could explore the impact of varying session lengths on cognitive outcomes in this population. Additionally, our study exclusively involved older adults from a single center. The inclusion of participants from multiple centers across different regions of Tunisia could have provided more diverse perspectives and potentially enriched the study outcomes. Furthermore, our study population was exclusively sourced from an urban setting, as individuals from rural areas were excluded. This exclusionary criterion may limit the generalizability of our results, and future studies could benefit from a more inclusive approach encompassing participants from diverse geographical backgrounds.

## 5. Conclusion

Twelve weeks of two 45-min yoga sessions per week significantly improved attention, memorization, and reasoning among older adults aged 65–80 years. These findings suggest that the benefits observed in this yoga program, conducted in a retirement home setting, may be applicable to similar facilities and populations of older adults with comparable characteristics. This highlights the potential of retirement home-based yoga classes as a cost-effective intervention for enhancing cognitive functions in aging populations.

For future research, it is recommended to explore larger and more diverse samples to further validate these findings across different demographic groups. Additionally, longer intervention durations could provide insights into the sustained benefits of yoga on cognitive health in older adults. Further investigation into specific cognitive domains, such as executive function and information processing speed, could also elucidate the broader impact of yoga interventions on cognitive aging.

## Figures and Tables

**Table 1:** Descriptive characteristics of the participants.

Groups	Yoga (*n* = 15; 6F)	APS (*n* = 15; 7F)	CONT (*n* = 15; 6F)
Age (years)	72.12 ± 3.35	73.25 ± 2.83	74.4 ± 3.55
Height (cm)	170.06 ± 5.82	169.51 ± 4.99	168.68 ± 5.21
Weight (kg)	72.74 ± 3.22	73.26 ± 4.57	73.13 ± 4.08
Sex (M/F)	9/6	8/7	9/6
Education level (P/S/U)	11/4/0	12/3/0	11/4/0
Baseline attention	12.87 ± 5.46	11.60 ± 4.37	8.80 ± 3.23
Baseline memory	4.60 ± 3.85	5.53 ± 3.58	3.40 ± 3.07
Baseline reasoning	5.47 ± 3.68	6.33 ± 4.12	5.67 ± 4.03

*Note:* Data are presented as mean ± standard deviation. Yoga: yoga group; APS: physical activity group; CONT: control group; F: female; P = primary; S = secondary; U = university.

## Data Availability

The data that support the findings of this study are available from the corresponding author upon reasonable request.

## Supporting Information

Additional file 1. CONSORT 2010 checklist of information to include when reporting a randomized trial∗.
